# Supplementary material for: Experimental evidence of mosaic structure in strongly supercooled molecular liquids
Source: Nat Commun. 2021 Mar 25;12:1867. doi: 10.1038/s41467-021-22154-8 (PMC7994800; doi:10.1038/s41467-021-22154-8)
Supplement: Supplementary file 1 — Supplementary Information [file 41467_2021_22154_MOESM1_ESM.pdf]

Supplementary Information: Experimental evidence of mosaic structure in strongly supercooled molecular liquids

F. Caporaletti,[1,*] S. Capaccioli,[2,3] S. Valenti,[4] M. Mikolasek,[5] A. I. Chumakov,[5,6] and G. Monaco[1,†]

[1] *Dipartimento di Fisica, Università di Trento, I-38123 Povo (Trento), Italy*

[2] *Dipartimento di Fisica, Università di Pisa, Largo Bruno Pontecorvo 3, I-56127 Pisa, Italy*

[3] *CISUP, Centro per l'Integrazione della Strumentazione dell'Università di Pisa, Lungarno Pacinotti 43, I-56127 Pisa, Italy*

[4] *Department of Physics, Universitat Politècnica de Catalunya, C. Jordi Girona, 1-3. 08034 Barcelona, Spain*

[5] *ESRF-The European Synchrotron, CS40 220, 38043 Grenoble Cedex 9, France*

[6] *National Research Center "Kurchatov Institute", 123182 Moscow, Russia*

* f.caporaletti@uva.nl; Current address: Van der Waals-Zeeman Institute, Institute of Physics/Van 't Hoff Institute for Molecular Sciences, University of Amsterdam, 1098XH Amsterdam, Netherlands

† giulio.monaco@unipd.it; Current address: Dipartimento di Fisica ed Astronomia, Universitá di Padova, I-35121 Padova, Italy

SUPPLEMENTERY NOTE 1. BROADBAND DIELECTRIC SPECTROSCOPY DATA.

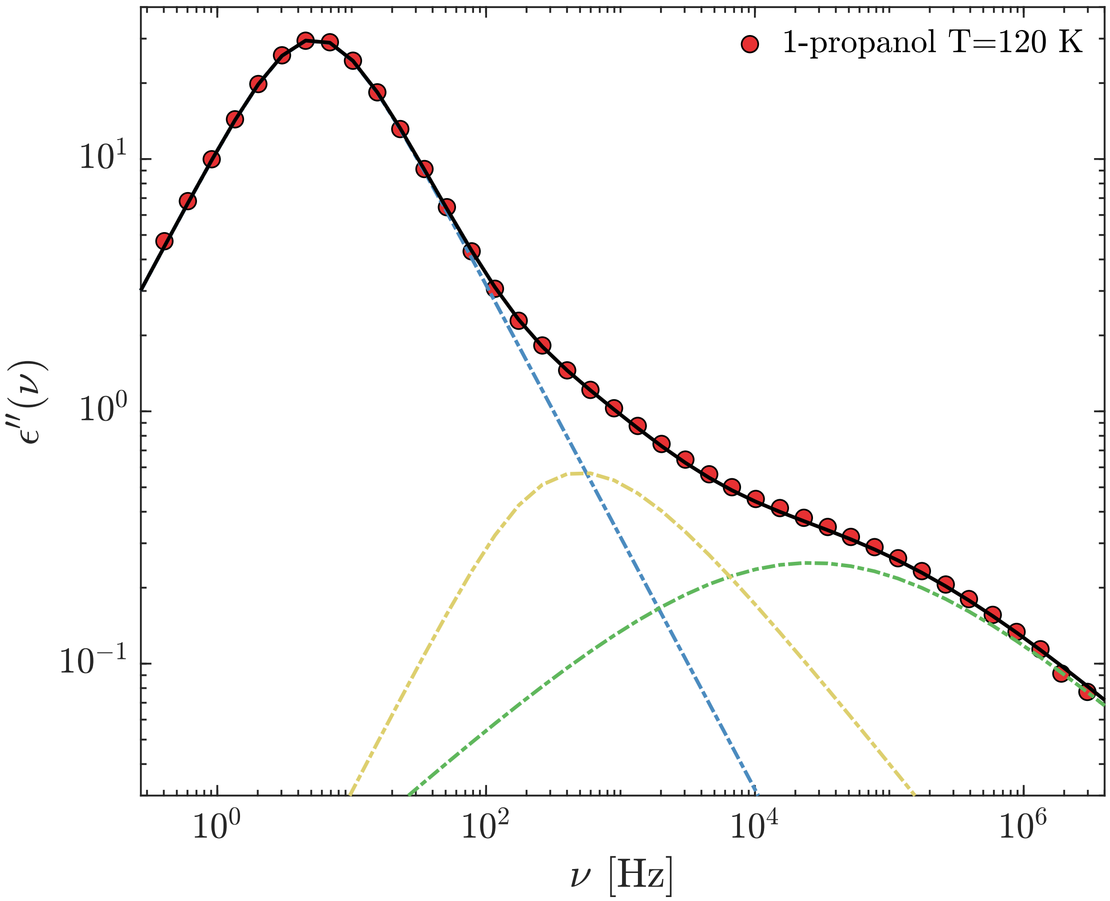

Supplementary Figure 1. **Dielectric loss spectrum of 1-propanol at T=120 K**. Red circles: experimental data; black solid line: full model curve fitted to the spectrum; blue dash-dotted line: Debye peak; yellow dash-dotted line: structural relaxation; green dash-dotted line: Johari-Goldstein relaxation.

The measured permittivity function $\epsilon(\nu)$ was analyzed fitting simultaneously its real $\epsilon'(\nu)$ and imaginary $\epsilon''(\nu)$ part, where ν is the frequency. The function used for the fits is reported in the Methods. An example of a dielectric spectroscopy loss spectrum of 1-propanol at T =120 K is reported in Supplementary Fig. 1 along with the curve obtained from the fitting procedure (black solid line) and the individual contributions of the Debye (blue dash-dotted line), α (yellow dash-dotted line) and β_{JG} (green dash-dotted line) relaxations.

SUPPLEMENTERY NOTE 2. COMPARISON BETWEEN NEUTRON SCATTERING AND TDI DATA.

Supplementary Fig. 2 reports the τ values extracted from the TDI beating patterns measured at q =15 and 25 nm^{-1} as in Fig. 2 along with the τ values measured by coherent quasi-elastic neutron scattering (QENS) at q =14 nm^{-1} [1] but at higher temperatures. The two data sets are clearly consistent.

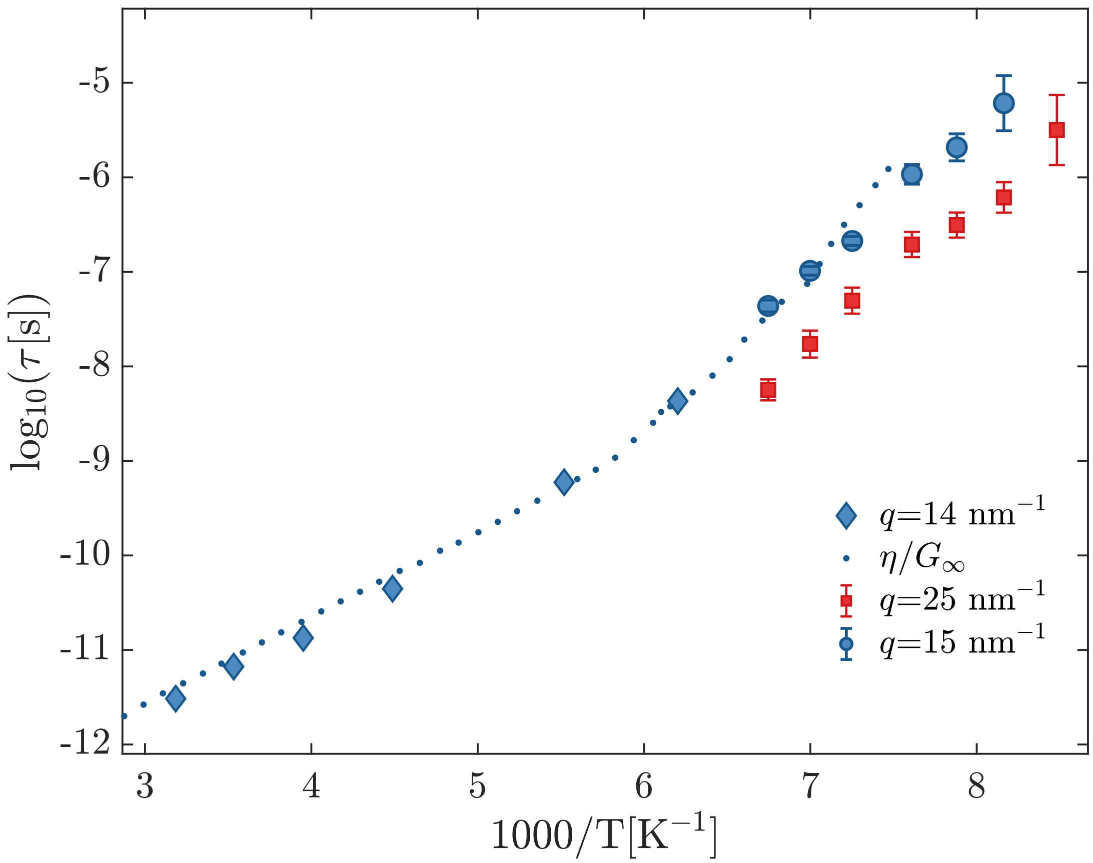

Supplementary Figure 2. **Comparison between QENS and TDI relaxation time data.** Blue circles and red squares with ±1 SD errorbars: TDI data, as in Fig. 2. Blue diamonds with ±1 SD errorbars: τ measured at $q_{\max}$ =14 nm^{-1} by QENS [1]. Dotted-line: scaled shear viscosity data, as reported in [1].

SUPPLEMENTERY NOTE 3. MEAN SQUARED DISPLACEMENT.

In this section the model used to quantitatively relate $q_{\rm DS}$ to the molecular mean-squared displacement, $\Delta r_{\rm JG}$, is discussed, where $q_{\rm DS}$ is the q-value at which the $\beta_{\rm JG}$ relaxation characteristic time appearing in the density correlation function matches the one from DS. The connection between $q_{\rm DS}$ and $\Delta r_{\rm JG}$ follows from an argument similar to the one at the basis of the anomalous diffusion model introduced in Ref. [2, 3] to describe sub-diffusion in polymers. The experimental observations for the $\beta_{\rm JG}$-process reported in the main text, as discussed in the following, are in fact up to a certain extent similar to those reported in [2], given the sub-diffusivity of the $\beta_{\rm JG}$-process. However, differently from what reported in [3], this comparison will be discussed here only in terms of the characteristic relaxation time probed by TDI and DS without any implication for the underlying distributions of relaxation times.

As a first point it can be noticed that density fluctuations within the $\beta_{\rm JG}$-relaxation, at least in the (reduced) time-window accessible to scattering techniques based on X-rays and neutrons, are well described by the KWW model [4–8]:

$$\phi(t) = f_q^{\rm JG} \exp\left[-\left(\frac{t}{\tau}\right)^{\beta_{\rm KWW}}\right] . \tag{1}$$

It is also important to point out that in a previous work on 5M2H [4] the KWW expression was found to be able to describe the $\beta_{\rm JG}$ relaxation, within experimental accuracy, even in a T and q range where the $\beta_{\rm JG}$-process i) dominates density fluctuations and ii) completely relaxes them within the time window accessed by TDI. The super-quadratic q-dependence of the $\beta_{\rm JG}$-relaxation characteristic time is a further aspect to consider:

$$\tau = A q^{-n} = \tilde{D}^{-\frac{n}{2}} q^{-n} \tag{2}$$

In Supplementary Eq. 2, $\tilde{D}$ can be regarded as a generalised diffusion coefficient with dimensions $[\tilde{D}] = [L]^2[t]^{-\frac{2}{n}}$ [3, 9]. If Supplementary Eq. 2 is combined with Supplementary Eq. 1, the intermediate scattering function can be written as:

$$\phi(q,t) = f_q^{\rm JG} \exp\left[-\left(\tilde{D}^{\frac{n}{2}} q^n t\right)^{\beta_{\rm KWW}}\right] \approx f_q^{\rm JG} \exp\left[-\tilde{D} q^2 t^{\beta_{\rm KWW}}\right] \tag{3}$$

The last approximation in Supplementary Eq. 3 is justified by the fact that, close to $q_{\rm DS}$, $n \cdot \beta_{\rm KWW} \approx 2$ for all the samples considered here within one standard deviation and with

Sample	T [K]	$\beta_{KWW}(q_{DS})$	n
5M2H	165.5	0.51	4.2(7)
5M2H	170.4	0.51	4.5(7)
1-propanol	122.5	0.66	3.9(9)
OTP	240	0.6	3.8(7)
OTP	265	0.6	2.9(5)

Supplementary Table I. **Stretching parameters and power-law exponents**. Stretching parameter β_{KWW} used in the fitting procedure of the TDI beating patterns for $q > q_{max}$ (and therefore valid at q_{DS}) for 1-propanol (present work), 5-methyl-2-hexanol (5M2H) [4] and o-terphenyl (OTP) [6, 7]. The power-law exponents (n) are also reported as obtained from fitting the q-dependencies of the characteristic time τ_p reported in the present work, recalculated from the data reported in Ref.[4] and taken from Refs.[6, 7].

deviations smaller than 30% (see Supplementary Fig. 3 and Supplementary Tab. I). It is also useful to recall another important result from previous work on 5M2H [4]: around q_{DS} (25 nm^{-1} < q <40 nm^{-1}) the strength of the β_{JG}-relaxation (f_q^{JG}) shows no q-dependence. Unfortunately no similar information is available for 1-propanol and o-terphenyl (OTP), as well as no information is available for the entire q-range where 5M2H was investigated. It is nonetheless reasonable to assume that around q_{DS} the relaxation strength does not show a strong q-dependence. Therefore Supplementary Eq. 3 shows approximately a Gaussian shape in q and it is therefore possible to re-write it as [10]:

$$\phi(q,t) \approx \exp\left[-\frac{\langle r^2(t)\rangle q^2}{6}\right] \tag{4}$$

and interpret $\langle r^2(t)\rangle$ as the mean-squared displacement of the molecule (MSD):

$$\langle r^2(t)\rangle = \langle|\mathbf{r}(t)-\mathbf{r}(0)|^2\rangle = 6\tilde{D}t^{2/n} \simeq 6\tilde{D}t^{\beta_{KWW}}. \tag{5}$$

It is important to remark that Supplementary Eq. 5 is consistent with the assumption that the incoherent approximation is valid. Namely, in the q-range here considered the contribution to the intermediate scattering function describing the β_{JG}-relaxation can be approximated with its self-part, and therefore collective molecular motions can be neglected: the β_{JG}-relaxation at times of the order of $\tau_{\beta_{JG}}^{DS}$ is essentially a single molecule process. This picture, valid at least in the explored q-range and before the onset of the α-relaxation, is

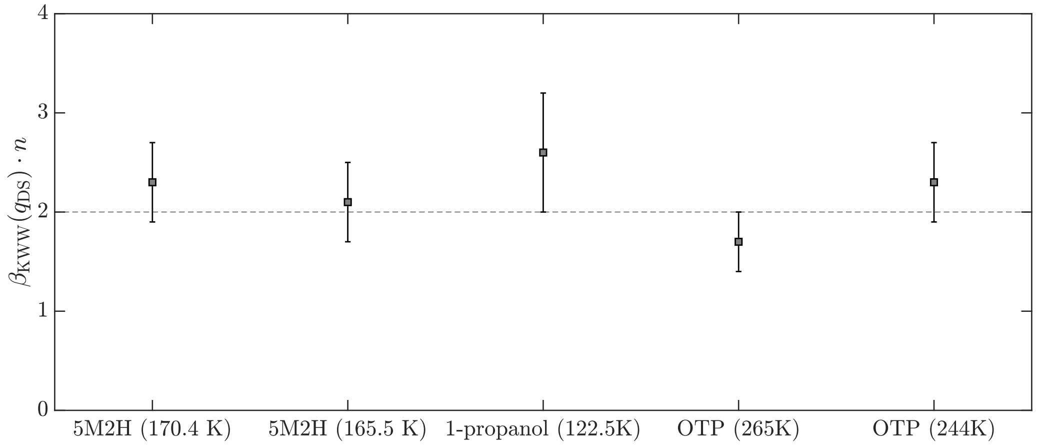

Supplementary Figure 3. **Justification for the description of the intermediate scattering function in terms of a Gaussian.** Product between the power law exponent (n) describing the q-dependence of the characteristic time of the β_{JG} relaxation and the stretching exponent $\beta_{\mathrm{KWW}}(q_{DS})$ for the three samples analysed in this work: 5-methyl-2-hexanol (5M2H), 1-propanol and o-terphenyl (OTP). The black dashed line indicates the value $n \cdot \beta_{\mathrm{KWW}} = 2$. The values of n and $\beta_{\mathrm{KWW}}(q_{DS})$ for 5M2H have been recalculated from the data reported in Ref. [4], and those for OTP are taken from Refs. [6, 7]. The plotted errorbars correspond to ± 1 SD.

consistent with two other observations: 1) the q-dependence of $\tau^{\mathrm{DS}}_{\beta_{\mathrm{JG}}}$ indicates a strongly restricted character of the process and 2) no de Gennes narrowing, hint of strong molecular correlations, was observed in the q-dependence of $\tau^{\mathrm{DS}}_{\beta_{\mathrm{JG}}}$. From Supplementary Eq. 5 it is then possible to extract the MSD at $\tau^{\mathrm{DS}}_{\beta_{\mathrm{JG}}}$:

$$\langle r^2(\tau^{\mathrm{DS}}_{\beta_{\mathrm{JG}}})\rangle = \frac{6}{q^2_{\mathrm{DS}}}\,. \tag{6}$$

We can finally use $\langle r^2(\tau^{\mathrm{DS}}_{\beta_{\mathrm{JG}}})\rangle$ to evaluate the average center-of-mass molecular root mean-squared displacement at the characteristic time of the β_{JG}-relaxation, $\tau^{\mathrm{DS}}_{\beta_{\mathrm{JG}}}$, as:

$$\Delta r_{\mathrm{JG}} = \sqrt{\frac{6}{q^2_{\mathrm{DS}}}}\,. \tag{7}$$

This expression gives a more solid basis to the previously used concept of typical length-scale for the β_{JG} process [4]. It is however important to stress that Δr_{JG} has to be considered as the most probable displacement of the molecules participating to the β_{JG}-relaxation at $\tau^{\mathrm{DS}}_{\beta_{\mathrm{JG}}}$. Since the relaxation strength of the β_{JG}-process is not known in the whole q-range, it

is not currently possible to estimate the full distribution of displacements associated to this relaxation process.

SUPPLEMENTERY NOTE 4. O-TERPHENYL: COMPARISON OF TDI AND DS DATA.

The OTP relaxation map from the TDI measurements reported in [6–8] is plotted in Supplementary Fig. 4, along with the characteristic times for the $\beta_{\rm JG}$-relaxation from DS [11] and adiabatic calorimetry (AC) [12] (see also the inset in Supplementary Fig. 4). As it can be observed in Supplementary Fig. 4, the $\beta_{\rm JG}$-relaxation decouples from the α one around 273 K. Differently from the case of 1-propanol and 5M2H [4], the $\beta_{\rm JG}$-relaxation was observed only at the intra-molecular length-scale [6–8]. It can be inferred that the activation energy of the $\beta_{\rm JG}$-relaxation as probed by the TDI data is quite consistent with the one from the DS/AC measurements extrapolated above Tg.

From Supplementary Fig. 4 it also clearly appears that molecular re-orientations within the $\beta_{\rm JG}$-process are faster than density fluctuations at q =18 nm^{-1} and slightly slower than those at 29 nm^{-1}, in line with what observed in 1-propanol and 5M2H. Concerning the q-dependence of the $\beta_{\rm JG}$-relaxation, a super-quadratic q-dependence was observed also in OTP [6, 7]: n =2.9(5) at 265 K and n =3.8(7) at 240 K. $q_{\rm DS}$ was then extracted from these q-dependent data in an analogous way as for 1-propanol and 5M2H (see main text). These $q_{\rm DS}$ data have then been used to extract the average center-of-mass displacement, $\Delta r_{\rm JG}$, at the characteristic time of the $\beta_{\rm JG}$-relaxation, see Supplementary Eq.7. The mean of the values obtained at 244 and 265 K (see Fig.4) and normalized to the average intermolecular distance is $\left\langle \frac{\Delta r_{\rm JG}}{r_{\rm p}} \right\rangle$ =0.125(17).

For the specific case of OTP, mean-squared displacement data are available for the crystalline phase at few temperatures [13]. It is then possible to extrapolate those data at the melting temperature, T_m=329 K: the obtained value is 0.604(9)Å^2, whose square root corresponds to 0.113(2) $r_{\rm p}^c$, where $r_{\rm p}^c$ is the average intermolecular distance estimated from the density of the crystal close to the melting temperature [14]. In the spirit of the Lindemann criterion, this could be considered as the limiting root mean-squared displacement for structural instability in OTP. It jumps to the eyes that this value coincides, within one SD, with the value reported above for $\left\langle \frac{\Delta r_{\rm JG}}{r_{\rm p}} \right\rangle$. This result, which could be verified here only for the case of OTP and

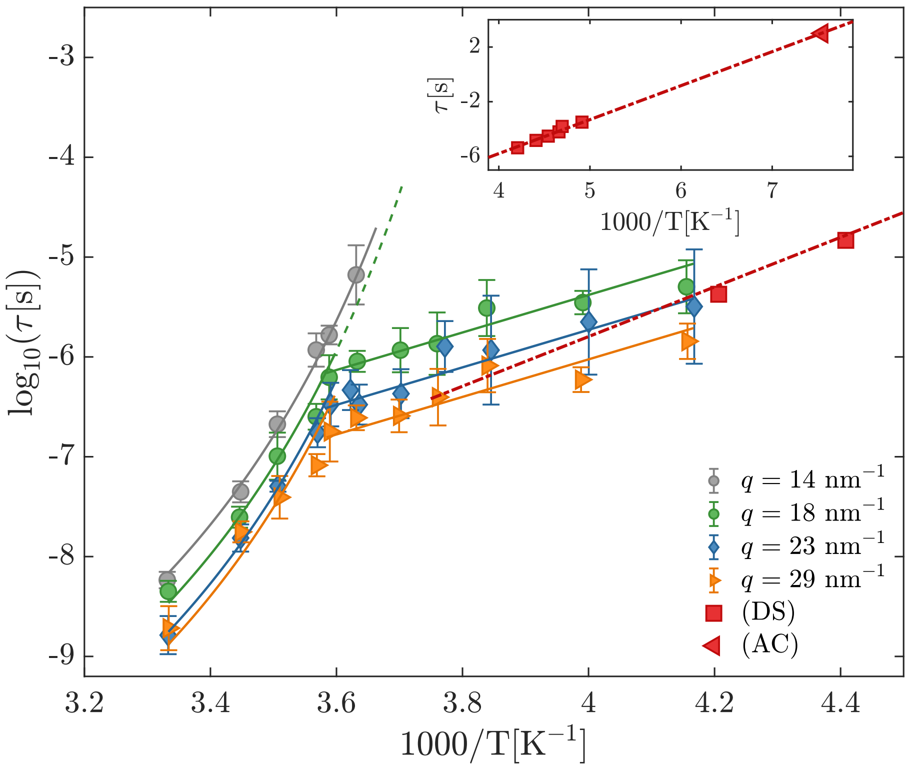

Supplementary Figure 4. **Relaxation map of o-terphenyl.** Inverse-temperature dependence of the relaxation time measured by TDI at different q-values: 14 nm^{-1} [6] (grey circles), 18 nm^{-1} [8] (green circles), 23 nm^{-1} [6] (blue diamonDS) and 29 nm^{-1} [8] (yellow right-pointing triangles). The solid lines of the corresponding color show the best fitting curves using the T-dependence for the α and β_{JG}-relaxation as reported in [6, 8]. The characteristic time of the β_{JG}-relaxation as measured by dielectric spectroscopy (DS) [11] (red squares) and adiabatic calorimetry (AC) [12] (red left-pointing triangles) are also reported for the sake of comparison. The red dash-dotted line is the Arrhenius fit to the DS and AC data. Inset: extended inverse-T dependence of the DS and AC data. All errorbars correspond to ±1 SD.

that might be fortuitous at this stage, implies that the average mean-square displacement at $\tau_{\beta_{\mathrm{JG}}}^{\mathrm{DS}}$ for the molecules participating to the β_{JG}-relaxation matches exactly the limiting value for structural instability (in terms of CM-CM distance), a conclusion stricter than the one reported in the main text. If we look at the β_{JG}-relaxation as a distribution of elementary processes with different timescales, all the processes relaxing with a characteristic time longer than $\tau_{\beta_{\mathrm{JG}}}^{\mathrm{DS}}$ satisfy then the Lindemann criterion for structural instability. It is however

important to underline that the preceding discussion has to be taken with a grain of salt, as the Lindemann limiting mean-square displacement extrapolated at the melting point might differ from the one for the amorphous structure of the glass. It is in fact known that the Lindemann limiting mean-squared displacement does depend on the local structure even for crystalline materials [15], and might therefore differ as well between the glass and the corresponding crystal.

SUPPLEMENTERY NOTE 5. NEAREST NEIGHBORS CALCULATIONS.

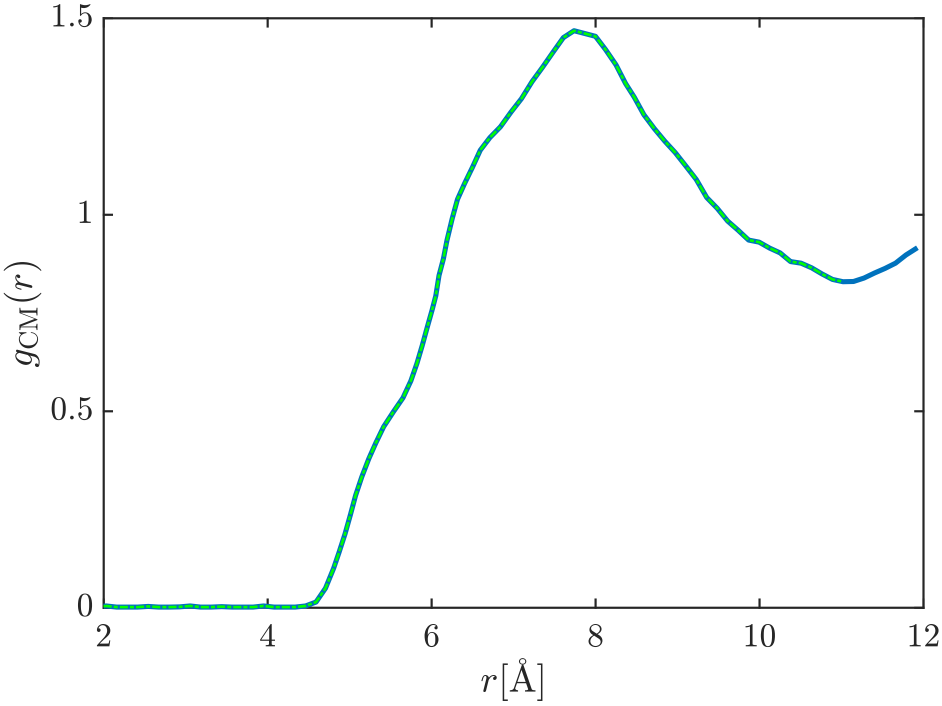

Supplementary Figure 5. **Center-of-mass pair distribution function.** Calculated molecular center-of-mass radial pair distribution function for o-terphenyl [17] at 300 K (blue solid line). The yellow dashed-line enlightens the range corresponding to the first coordination shell.

The average coordination number z can be easily estimated for 1-propanol and OTP integrating their radial intermolecular pair distribution functions, $g(r)$, over the first coordination shell [16]:

$$z = \int_0^{r_{min}} 4\pi\rho r^2 g(r) dr \,. \tag{8}$$

Here r_{min} marks the position of the first minimum in $g(r)$, and ρ is the number density. In the case of OTP the radial distribution function relative to the molecular center-of-mass (g_{CM}) (see Supplementary Fig. 5) was used, taken from Ref. [17]. In the case of 1-propanol, given its more complex structure, the partial $g(r)$ corresponding to inter-molecular correlations between the central carbon atoms of each alkyl-chain was employed instead (D. Bowron, private communication). Both $g(r)$ datasets correspond to room temperature, T =300 K. The obtained z values are reported and discussed in the main text. These values are not expected to vary by more than 20% at the lower temperatures where the TDI and DS experiments were carried out, based on the corresponding changes in the density of the two samples (see Refs. [17] and [18]).

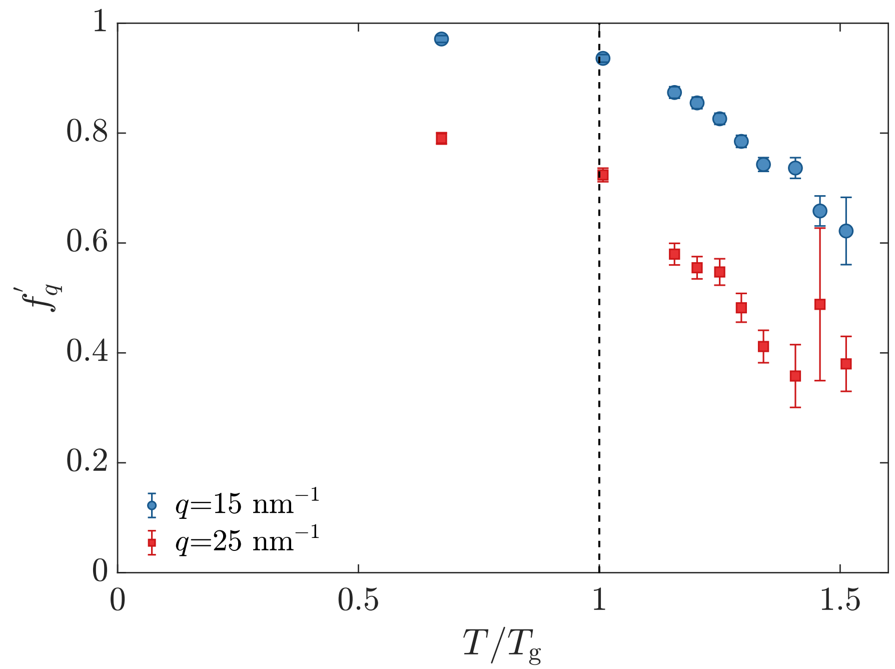

Supplementary Figure 6. **Tg-scaled temperature dependence of the initial beating contrast of the TDI signal.** The initial beating pattern f'_q is plotted as measured at two different exchanged wave-vectors: 15 (blue circles with ±1 SD errorbars) and 25 nm^{-1} (red squares with ±1 SD errorbars).

SUPPLEMENTERY NOTE 6. INITIAL BEATING PATTERN CONTRAST.

A. T-dependence

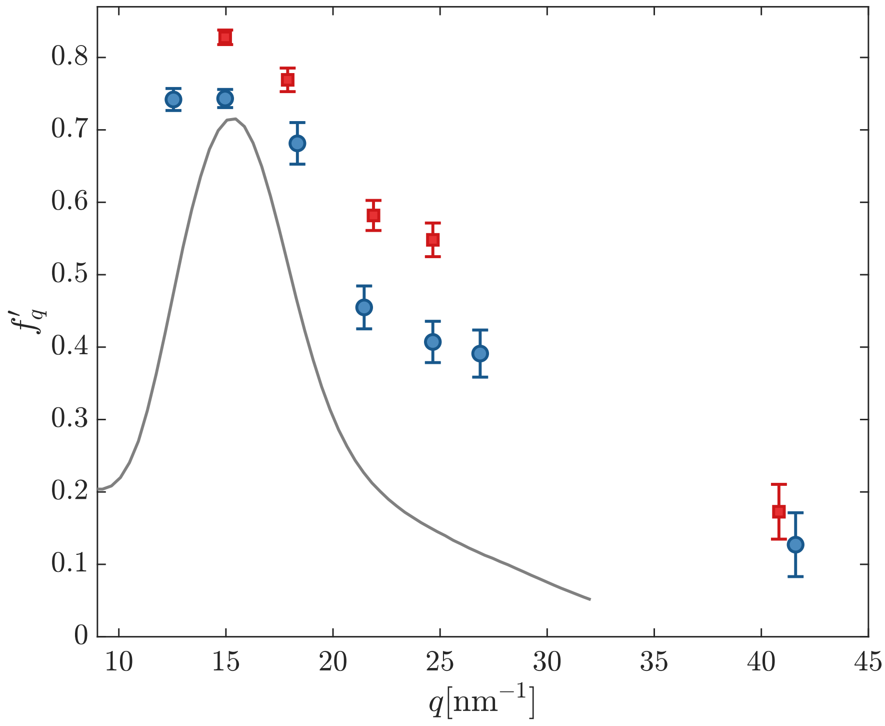

Supplementary Figure 7. **Wave-number dependence of the initial beating pattern contrast.** The q-dependence of the initial beating pattern f'_q is plotted as measured at two different temperatures: T=131.4 (blue circles with ±1 SD errorbars) and 122.5 K (red squares with ±1 SD errorbars). The diffuse scattering pattern measured at T=122.5 K is rescaled and reported on the same axes for sake of comparison.

The initial beating contrast, f'_q, corresponding to the τ values reported in Fig. 2 are shown in Supplementary Fig. 6 along with the initial beating contrast values extracted at lower temperatures, where the dynamics is too slow to be probed by TDI. f'_q is related to the strength of the α-relaxation process above $T_{\alpha\beta} \simeq$131 K whereas below this temperature it accounts for the relaxation strength of both the α and the β_{JG}-relaxation. In fact for $T < T_{\alpha\beta}$ the dynamics is too slow to observe a complete decorrelation in the time window accessed by TDI and the relative contributions of the β_{JG} and α-processes cannot be disentangled. It can be noticed that, consistently with what already observed in a previous TDI study

of 5-methyl-2-hexanol [4], f'_q does not show any discontinuity at $T_{\alpha\beta}$, meaning that when the β_{JG}-process separates from the α-one the total relaxation strength is conserved. The T-dependence of f'_q shows instead a clear change in slope at Tg.

B. q-dependence

The q-dependence of the initial beating contrast, f'_q, corresponding to the τ values reported in Fig. 3 is plotted in Supplementary Fig. 7. At both probed temperatures, T =131.4 (blue circles with $\pm$1 SD errorbars) and 122.5 K (red squares with $\pm$1 SD errorbars), f'_q displays an oscillation in phase with the $S(q)$ of 1-propanol. This oscillation can be associated to the well-known de Gennes narrowing effect [19–21] and signals the sensitivity to intermolecular correlations of the process(es) contributing to f'_q. As already discussed in the main text, the TDI data are sensitive to the faster relaxation process and to the total strength.

SUPPLEMETARY REFERENCES

[1] Sillrén, P. et al. Liquid 1-propanol studied by neutron scattering, near-infrared, and dielectric spectroscopy. *J. Chem. Phys.* **140**, 124501 (2014).

[2] Colmenero, J., Alegría, A., Arbe A. & Frick, B. Correlation between non-Debye behavior and Q behavior of the α relaxation in glass-forming polymeric systems *Phys. Rev. Lett.* **69**, 478-481 (1992).

[3] Arbe, A. & Colmenero, J. *The Scaling of Relaxation Processes.* (Springer, Berlin, Heidelberg, 2018); pp. 247-245.

[4] Caporaletti, F. et al. A microscopic look at the Johari-Goldstein relaxation in a hydrogen-bonded glass-former. *Sci. Rep.* **9**, 14319 (2019).

[5] Arbe, A., Buchenau, U., Willner, L., Richter, D., Farago, B. & Colmenero, J. Study of the dynamic structure factor in the β-relaxation regime of polybutadiene. *Phys. Rev. Lett.* **76**, 1872-1875 (1996).

[6] Saito, M. et al. M. Slow Processes in Supercooled o-Terphenyl: Relaxation and Decoupling. *Phys. Rev. Lett.* **109**, 115705 (2012).

[7] Saito, M. et al. Slow dynamics of supercooled liquid revealed by Rayleigh scattering of Mössbauer radiation method in time domain. *Hyp. Int.* **226**, 629-636 (2014).

[8] Kanaya, T., Inoue, R., Saito, M., Seto, M. & Yoda, Y. Relaxation transition in glass-forming polybutadiene as revealed by nuclear resonance X-ray scattering. *J. Chem. Phys.* **140**, 144906 (2014).

[9] Metzler. R & Klafter, J. The random walk's guide to anomalous diffusion: a fractional dynamics approach. *Phys. Rep.* **339**,, 1-77 (2000).

[10] J.-P. Hansen & McDonald, I.R. *Theory of simple liquiDS.* (Academic, London, 1986).

[11] Johari, G. P. & Goldstein, M. Viscous Liquids and the Glass Transition. II. Secondary Relaxations in Glasses of Rigid Molecules. *J. Chem. Phys.* **53**, 2372-2388 (1970).

[12] Fujimori, H. & Oguni, M. Correlation index $(T_{g\alpha}\text{-}T_{g\beta})/T_{g\alpha}$ and activation energy ratio as parameters characterizing the structure of liquid and glass. *Solid State Commun.* **94**, 157-162 (1995).

[13] Tölle, A., Zimmermann, H., Fujara, F., Petry, W., Schmidt, W., Schober, H. & Wuttke, J. Vibrational states of glassy and crystalline orthoterphenyl. *Eur. Phys. J. B* **16**, 73 (2000).

[14] Greet, R. J. & Turnbull, D. Glass Transition in oTerphenyl. *The Journal of Chemical Physics* **46**, 1243 (1967);

[15] Cho S.-A.,Role of lattice structure on the Lindemann fusion theory of metals . *J. Phys. F: Met. Phys.* **12**, 1069 (1982)

[16] Brostow, W. Radial distribution function peaks and coordination numbers in liquiDS and amorphous solids. *Chem. Phys. Lett.* **49**, 285-288 (1977).

[17] Mossa, S., Di Leonardo, R., Ruocco, G. & Sampoli, M. Molecular dynamics simulation of the fragile glass-former orthoterphenyl: A flexible molecule model. *Phys. Rev. E* **62**, 612 (2000).

[18] Zakaryaev, Z. R. Experimental determination of propanol-1 P, ρ, T data. *J. Eng. Phys.* **43**, 1261–1262 (1982).

[19] de Gennes, P. G. Liquid dynamics and inelastic scattering of neutrons. *Physica* **25**, 825-839 (1959).

[20] Tölle A., Neutron scattering studies of the model glass former ortho-terphenyl. *Rep. Prog. Phys.* **64**, 1473-1532 (2001).

[21] Sciortino, F., Fabbian, L., Chen, S.-H. & Tartaglia, P. Supercooled water and the kinetic glass transition. II. Collective dynamics. *Phys. Rev. E* **56**, 5397-5404 (1997).
